# Improving Case Definitions for Severe Malaria

**DOI:** 10.1371/journal.pmed.0040267

**Published:** 2007-08-21

**Authors:** Nicholas M Anstey, Ric N Price

## Abstract

The authors discuss a study by Philip Bejon et al. that adopted a new approach to balancing the sensitivity and specificity of criteria for defining severe malaria.

The lack of a “gold standard” definition for severe malaria has been a longstanding problem for both clinicians and researchers. The definitions currently used comprise a set of clinical and laboratory parameters associated with an increased risk of death [1,2], combined with the presence of Plasmodium falciparum parasitemia [2,3]. In young children, these criteria are predominantly altered consciousness, severe anemia, and respiratory distress [1,3]; a broader range of criteria is applicable to adolescents and adults [2].

While these criteria are sensitive in diagnosing severe malaria, they are also present in other serious illnesses. Since asymptomatic parasitemia is common in malaria-endemic areas, patients fulfilling current World Health Organization (WHO) criteria for severe malaria [3] often have disease attributable to another cause, such as bacterial sepsis with incidental parasitemia [4], thereby limiting the specificity of this definition.

For a treating clinician, a sensitive but less specific definition of severe malaria is entirely appropriate. However, for research purposes, a sensitive clinical definition may not necessarily be appropriate, and the case definition should depend on the research question.

For example, in a recent randomized controlled trial of quinine versus artesunate for severe malaria [5], the aim was to compare the efficacy of two drugs in real-world resource-poor clinical settings. The primary endpoint, mortality, included all patients enrolled with a bedside clinical diagnosis of severe malaria, who were positive for P. falciparum by a rapid diagnostic test. This sensitive but less specific definition had clinical applicability, but necessitated a large sample size to overcome the loss of power from the inclusion of patients with less severe disease or severe illness unrelated to malaria.

At the other end of the spectrum, studies of severe malaria pathophysiology require definitions with much higher specificity to ensure accurate identification of mechanisms of disease; sensitivity is less important in these situations. Such specificity in severe malaria has been improved using extended laboratory criteria, lumbar puncture, indirect ophthalmoscopy [6], autopsy validation [7], and a high parasite density threshold [2,8].

**Linked Research Article**

This Perspective discusses the following new study published in *PLoS Medicine:*

Bejon P, Berkley JA, Mwangi T, Ogada E, Mwangi I, et al. (2007) Defining childhood severe falciparum malaria for intervention studies. PLoS Med 4: e251. doi:10.1371/journal.pmed.0040251

The accepted definition of severe malaria is appropriate for clinical purposes, but Philip Bejon and colleagues show that its specificity in clinical trials may be improved by applying a parasite density threshold and by excluding children with certain conditions.

## Defining Severe Malaria Cases in Community Intervention Studies

What definition for severe malaria should be used when severe malaria is a study endpoint rather than an entry criterion? In vaccine trials or intervention studies designed to prevent severe disease, case definitions should reflect a balance between sensitivity and specificity. Neither of the definitions described above would be optimal. Ideally, a level of specificity would be adopted that ensures an accurate estimate of vaccine efficacy, without reducing the sensitivity to a level that requires a major increase in the sample size.

In this issue of *PLoS Medicine*, Philip Bejon et al. present an important study in which they adopt a new approach to balancing the sensitivity and specificity of criteria for severe malaria [9]. The authors define malaria attributable fractions (MAFs) [10] for different case definitions of severe malaria in children in Kenya, which estimate the positive predictive value for severe malaria for a given background prevalence of asymptomatic parasitemia, i.e., the proportion of severe disease with parasitemia that is attributable to malaria.

Using a sample of 4,583 community-based children with asymptomatic parasitemia and 1,422 hospitalized children with signs of severe malaria, the risk of severe disease was modeled using logistic regression with varying parasite densities and a range of comorbidities that may have altered the specificity of their definition of severe malaria. In their coastal Kenyan setting, they calculated that an overall MAF of 95% could be achieved by applying a threshold parasitemia of more than 2,500 parasites/μl after excluding children with meningitis, clinician-diagnosed lower respiratory infection, bacteremia, and gastroenteritis with severe dehydration.

The authors' rigorous analysis shows what can be achieved with large datasets derived from meticulous prospective collection of clinical and laboratory information. The study included data on asymptomatic parasitemia from settings with different transmission intensity. The researchers were able to collect blood cultures from all hospitalized children, and HIV status was available for almost all.

Despite the strengths of the study, there are some limitations. The absence of indirect ophthalmoscopy [6] (although this is not a widely available procedure) and of cerebrospinal fluid examination in 22% of children with signs of cerebral malaria may have reduced the positive predictive values of their definition. A MAF of 32% for children with high parasitemia and confirmed meningitis could imply a contributory role of malaria to meningitis, and vice versa, but could also indicate some inaccuracy of the MAF in estimating the true burden of severe malaria.

Given the high prevalence of HIV infection in African children and known malaria–HIV interactions, a significant finding was that HIV and malnutrition did not reduce the MAF, leading the authors to recommend that such patients should not be excluded from the case definition. Interestingly, the MAF for respiratory distress defined by deep breathing [1] was the same as that for the potentially more objective measure of raised respiratory rate. Other notable findings included the high predictive value for severe malaria of gram-negative bacteremia (plausibly secondary to malaria) and low positive predictive value for gram-positive bacteremia (plausibly incidental to malaria).

## Clinical Implications and Future Research

Bejon et al. have now demonstrated the utility of such methodology in identifying a case definition that appropriately balances sensitivity and specificity for severe malaria in coastal Kenya. Their definition provides a rational endpoint for future vaccine trials and other community-based malaria prevention trials at this site. The authors acknowledge that these findings are applicable to coastal Kenya and cannot necessarily be generalized to other settings. Nevertheless, the approach they have used will pave the way for others to derive locally applicable MAFs and test the generalizability of their findings. Indeed the derivation of MAFs should, where possible, become an intrinsic part of the planning of vaccine/intervention trials and epidemiology studies of severe malaria.

In areas of high malaria transmission, the parasitemic thresholds for defining severe disease will increase. Interestingly, in Kilifi, the thresholds defining uncomplicated and severe malaria were similar. If confirmed elsewhere, this raises the possibility that when parasite density thresholds are available for uncomplicated malaria [10] but not for severe disease, the former could potentially be applied to the latter.

Recent studies suggest that 30% of falciparum malaria occurs outside Africa, mostly in South and Southeast Asia [11], and often in areas with lower, unstable transmission where severe disease occurs in adults as well as children. Calculation of MAFs will be required for each of these age groups. In these regions P. vivax may account for more than 50% of infections and is a likely cause of a significant yet neglected burden of severe disease, particularly severe anemia in children [12]. A similar approach to defining attributable fractions for P. vivax infections as that demonstrated for P. falciparum will allow a better appreciation of the relative burden of P. vivax and other malaria species whose association with severe morbidity is often dismissed [12].

Finally, while the lengthy descriptions of severe malaria included in current WHO guidelines [3] are appropriate for clinical purposes, they lack clarity for research purposes. This is particularly true for case definitions in adults, resulting in the frequent use of “*modified* WHO criteria” to define severe malaria in published studies [2,5,13]. A parasite threshold to improve specificity in severe anemia was defined for research purposes in the 1990 WHO guidelines [8], but was dropped from the current WHO guidelines [3]. Forthcoming guidelines should more clearly and concisely establish case definitions for severe malaria in both children and adults, suitable for different research purposes in different epidemiological settings. For malaria prevention trials and disease burden studies, forthcoming WHO guidelines should highlight the utility of Bejon et al.'s MAF approach in developing locally applicable case definitions of severe malaria.

## Abbreviations

MAF: malaria attributable fraction
WHO: World Health Organization
